# Fast body part segmentation and tracking of neonatal video data using deep learning

**DOI:** 10.1007/s11517-020-02251-4

**Published:** 2020-10-23

**Authors:** Christoph Hoog Antink, Joana Carlos Mesquita Ferreira, Michael Paul, Simon Lyra, Konrad Heimann, Srinivasa Karthik, Jayaraj Joseph, Kumutha Jayaraman, Thorsten Orlikowsky, Mohanasankar Sivaprakasam, Steffen Leonhardt

**Affiliations:** 1 Medical Information Technology (MedIT), Helmholtz-Institute for Biomedical Engineering, RWTH Aachen University, Pauwelsstr. 20, 52074 Aachen, Germany; 2 Section of Neonatology, RWTH Aachen University, Pauwelsstr. 30, 52074 Aachen, Germany; 3 Department of Electrical Engineering, Indian Institute of Technology, Madras, Chennai, 600036 Tamil Nadu India; 4 Saveetha Medical College, Kanchipuram, Saveetha Nagar, Chennai, 602 105 India

**Keywords:** Image processing, Deep learning, Semantic segmentation, Camera-based monitoring, Nicu

## Abstract

Photoplethysmography imaging (PPGI) for non-contact monitoring of preterm infants in the neonatal intensive care unit (NICU) is a promising technology, as it could reduce medical adhesive-related skin injuries and associated complications. For practical implementations of PPGI, a region of interest has to be detected automatically in real time. As the neonates’ body proportions differ significantly from adults, existing approaches may not be used in a straightforward way, and color-based skin detection requires RGB data, thus prohibiting the use of less-intrusive near-infrared (NIR) acquisition. In this paper, we present a deep learning-based method for segmentation of neonatal video data. We augmented an existing encoder-decoder semantic segmentation method with a modified version of the ResNet-50 encoder. This reduced the computational time by a factor of 7.5, so that 30 frames per second can be processed at 960 × 576 pixels. The method was developed and optimized on publicly available databases with segmentation data from adults. For evaluation, a comprehensive dataset consisting of RGB and NIR video recordings from 29 neonates with various skin tones recorded in two NICUs in Germany and India was used. From all recordings, 643 frames were manually segmented. After pre-training the model on the public adult data, parts of the neonatal data were used for additional learning and left-out neonates are used for cross-validated evaluation. On the RGB data, the head is segmented well (82% intersection over union, 88% accuracy), and performance is comparable with those achieved on large, public, non-neonatal datasets. On the other hand, performance on the NIR data was inferior. By employing data augmentation to generate additional virtual NIR data for training, results could be improved and the head could be segmented with 62% intersection over union and 65% accuracy. The method is in theory capable of performing segmentation in real time and thus it may provide a useful tool for future PPGI applications.

**Graphical Abstract:**
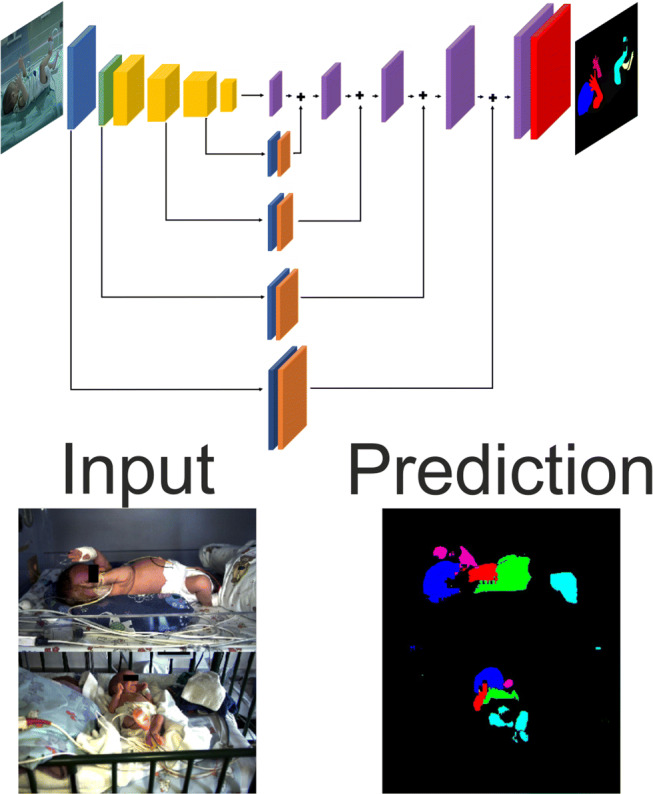
This work presents the development of a customized, real-time capable Deep Learning architecture for segmenting of neonatal videos recorded in the intensive care unit. In addition to hand-annotated data, transfer learning is exploited to improve performance.

This work presents the development of a customized, real-time capable Deep Learning architecture for segmenting of neonatal videos recorded in the intensive care unit. In addition to hand-annotated data, transfer learning is exploited to improve performance.

## Introduction

According to the World Health Organization, 15 million babies [1] are born prematurely each year and thus lack a fully developed biological and physiological system. Besides the neurodevelopmental problems that are highly associated with this type of patients, the functional immaturity of the various organs and their regularization mechanisms commonly lead to complications [2]. These can result in irregular cardiorespiratory patterns which can lead to clinical complications [3]. It is therefore crucial to perform continuous monitoring of cardiovascular signals as changes are often observed prior to major complications. State-of-the-art physiological monitoring of neonates involves skin-attached sensors, e.g., electrocardiography (ECG) electrodes, pulse oximeters, or temperature probes in combination with the respective wires. In addition to discomfort, contact-based sensors imply the risk of injuries, such as “medical adhesive-related skin injuries” (MARSI) which is a serious problem for preterm infants patients with vulnerable and fragile skin [4].

Camera-based monitoring technologies such as photoplethysmography imaging (PPGI) offer promising alternatives, as they allow remote estimation of heart rate (HR) and respiratory rate [5–7]. Although the precise origin of the cardiac-associated signal of PPGI is still being researched, the fundamental principle is the same for PPGI as it is for contact-based photoplethysmography (PPG): the pulsatile changes in blood pressure lead to rhythmic changes in the optical properties of the skin, which can be detected using single-point sensors in close contact with the skin (PPG), or, in case of PPGI, by using a camera-based sensing setup.

Considering the benefits of PPGI, monitoring of neonates is a particularly useful application scenario which has been addressed by several research groups. As of today, present results are based on relatively small datasets that range from seven [8] to 30 [9] different subjects. The videos were recorded either through the incubator glass [8, 10–12], directly with open incubators [13], or through a specially drilled hole in a closed incubator [9, 14]. Some dataset’s recordings comprise the majority of the infant’s body, whose skin is partially or not covered [14], while others apply zoom to focus specific uncovered body parts [13]. On the other hand, Sikdar et al. [15] present HR results based on a dataset that comprises a diverse range of body positions and angles with respect to the video camera. Maintaining the circadian rhythm of neonates is of major concern in the neonatal intensive care unit (NICU). Thus, when deploying PPGI technology in such an environment, the usage of invisible near infrared (NIR) illumination is desirable [10].

Tracking regions of interest (ROIs) remains to be a challenge. Despite presenting important contributions to the field regarding signal processing methods, in [8, 11–15], the PPGI signal was extracted within a manually chosen region that in some works is tracked along the frames resorting to rudimentary object tracking methods. Since the selected ROI contains merely skin, it offers only few recognizable image features, making it extremely difficult to track along the video frames. To address this challenge, researchers are developing new methods for automatic and continuous ROI selection that do not resort to human supervision.

In [3], the ROI selection is accomplished through a color-based 2-class classifier based on Gaussian Mixture models. It clusters each pixel from each frame into skin and non-skin classes. In this model, the ROI consists of the largest continuous skin region in each frame. However, having a color-based skin classifier, where the ROI is not associated with the anatomical structure of interest, leads to non-robust vital parameter extraction in continuous monitoring over extended periods of time, as the author states in his conclusions. Blanik et al. [10] opted to divide the video frames into squares of 30 pixels edge length and compute a quality index (QI) for each one. All the squares possessing a QI above a threshold of 90% of the maximum QI value will belong to the ROI and, consequently, will be used for HR estimation. Despite the fact that the ROI is not directly linked to an anatomical structure, this method guarantees that the selected area is representative of the parameter that will be extracted. However, in periods of intense motion, this technique still yields poor results.

Recently, methods from the realm of deep learning (DL) have gained enormous interest in virtually every image processing domain, including medical applications. For the scope of this work, we will use a broad definition of DL as structures of artificial neural networks with more than three layers. In particular for tasks such as classification and semantic image segmentation, DL has outperformed existing approaches. However, these gains could mainly be achieved if large annotated datasets are available for training. To some extended, the problem is alleviated by data augmentation, which creates manipulated copies of the training data to increase robustness and generalization of the training process. Nevertheless, annotated medical image datasets, in particular those obtained with technologies such as PPGI, which are not yet clinically established, are comparatively small.

Chaichulee et al. [9, 16] managed to detect the presence of the neonate in the incubator, identify the skin region, and define two different ROI for vital-sign estimation using DL. For this purpose, Chaichulee et al. proposed a convolutional neural network (CNN) with three branches from a shared core network. The patient detection branch was implemented using global average pooling with two outputs containing the prediction of the two classes. The skin segmentation branch was implemented following the “fully convolutional neural network” (FCNN) proposed by Long et al. [17]. The body part detection branch locates the neonate’s head, torso, and diaper relying on bounding boxes using a faster “region-based convolutional network” R-CNN network [18]. The model performs patient detection with 98.75% accuracy, 97.56% precision, and 100% recall. In terms of skin segmentation, a mean pixel accuracy of 98.05 % and a mean Intersection over Union (IoU) of 88.57 % is achieved. The authors reported a mean absolute error of 2.4 beats per minute for 80% of recording time in terms of HR estimation. However, as the authors state in the discussion section, the model is unable to achieve real-time performance given its “VGG-16” feature extractor [19] and its region proposal generation network.

Also outside of the NICU setting, human body part segmentation is a challenge in the computer vision community, and large datasets exist: For example, the PASCAL human parts dataset is a subset of the general PASCAL VOC 2010 dataset [20], which contains extra detailed annotations of human body parts (eyes, nose, upper arm, etc.). The FCNN model proposed by Oliveira et al. [21] is designed to address the human body part segmentation problem relying on a less complex decoder network compared with previous work [22–24]. For the “PASCAL 4 parts dataset,” they report a mean accuracy of 76.58% and a mean IoU of 63.03%. The corresponding values for “PASCAL 14 parts” are 77%/54.18% and 88.2%/71.71% for an augmented dataset. For the “Freiburg People in Disaster” dataset, a mean IoU of 71.99% is reported. Similarly to [17] (mean IoU 62.7%/62.2% for PASCAL VOC 2011/2012), the encoder network of Oliveira et al. corresponds to a modified VGG-16 image classification network combined with a novel upsampling process.

In this work, we focus on the task of body part segmentation and tracking of neonates in the NICU. The goals of our approach are to develop a DL-based segmentation system:

- with real-time applicability based on the works by Oliveira et al. but with a less resource-demanding encoding stage
- which can deal with the problem of limited training data by exploring pre-training using publicly available datasets
- and is capable to process NIR data.

## Materials and methods

In the following, the dataset as well as algorithm used for segmentation is described.

### Data description

A dataset recorded in two different hospital settings was used in this work. The first subset was recorded at RWTH Aachen University Hospital (UKA), Department of Neonatology, Aachen, Germany (Aachen subset), and the study was approved by the ethics committee of the UKA, Aachen, Germany (EK 327/16). Nine neonates were placed in incubators or on warming beds/cribs. RGB data was recorded using the CMOS color camera GS3-U3-23S6C-C (FLIR, USA). NIR data was recorded using the monochrome CMOS camera GS3-U3-23S6M-C (FLIR, USA) equipped with a 940-nm filter (BN940, Midwest Optical Systems, Inc., USA). Images were recorded at *f*_s_ = 25 Hz and a shutter time of 19.5 ms at a resolution of 1920 × 1200, downsampled to 960 × 600 and cropped to 960 × 576, which ensures divisibility by 32. In addition to ambient light, a S75-WHI NIR lighting module at 940 nm (Smart Vision Lights, USA) was used.

The second subset (Chennai subset) was recorded at Saveetha Medical College and Hospital, Chennai, India, and the study was approved by the institutional ethics committee of Saveetha University (SMC/IEC/2018/03/067). Twenty neonates were recorded either under an infant radiant warmer or in a transport incubator [25]. The same cameras as in the Aachen subset were used. While illumination in the visual domain was ambient, active NIR illumination was provided using a matching LED lamp (S75-940-W, Smart Vision Lights, USA) and two layers of an additional diffusion filter (LEE Filters, UK).

In both datasets, the majority of the infants were awake during the whole measurements. Consequently, the recordings comprise a high level of motion. Only in seven recordings, minimal motion was present indicating calmly sleeping babies. Also, no constraints were imposed regarding the neonates’ position/orientation and clinical staff activity which proceeded normally with the patient care routine.

**Fig. 1:**
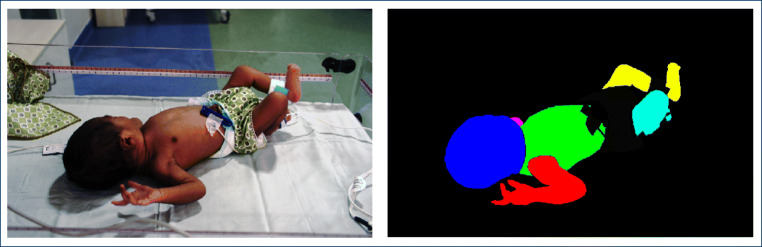
Ground-truth six-part segmentation: head (blue), torso (green), right arm (red), left arm (magenta), right leg (cyan), left leg (yellow). Note that only naked skin is segmented. White balance was corrected for visualization purposes

To generate segmented data for training and test, manual segmentation was performed using the MATLAB segmentation tool “Image Labeler” (The MathWorks, Inc., Natick, MA, USA). Each image was segmented into six parts, namely “head,” “torso,” “left arm,” “right arm,” “left leg,” and “right leg.” Only naked skin was segmented, i.e., occlusions due to clothing, electrodes, bandages, etc. were excluded. The process is visualized in Fig. 1.

To identify “interesting” frames for segmentation, a straightforward algorithm computed differences of consecutive frames. As lighting conditions were relatively constant, larger differences indicated movement. Using calculated differences as guidance, frames were manually selected for annotation. In total, 563 RGB and 80 NIR images were manually annotated.

To evaluate our approach, fivefold cross-validation was performed. Images were assigned to folds so that no images from one recording were distributed over multiple folds, i.e., complete recordings are left out for evaluation. This implies that complete subjects are left out in the evaluation process. In the dataset, skin tone of neonates varied substantially. While neonates from Europe tend to have relatively light skin, South Indian neonates tend to have darker skin, whereas North Indian skin tones usually fall somewhere in between. Moreover, neonates could be oriented “prone,” “supine,” or “on the side.” Thus, recordings were optimized to generate folds that are balanced in terms of frames, origin, and orientation for RGB and NIR frames. The resulting distribution is provided in Table 1.

**Table 1:** Summary of the dataset frame distribution in the fivefolds. *Su* supine, *Pr* prone, *Si* side, *Eu* European, *No* North Indian, *So* South Indian

Fold	RGB dataset frames							NIR dataset frames						
	Total	Per orientation			Per origin			Total	Per orientation			Per origin		
		Pr	Su	Si	Eu	No	So		Pr	Su	Si	Eu	No	So
1	106	3	103	0	30	14	62	19	1	18	0	5	2	12
2	121	14	107	0	38	18	65	17	4	13	0	8	2	7
3	109	12	97	0	34	14	61	13	2	11	0	5	1	7
4	121	0	113	8	30	23	68	15	0	14	1	3	3	9
5	106	0	106	0	40	5	61	16	0	16	0	3	1	12
Sum	563	29	526	8	172	74	317	80	7	72	1	24	9	47

### Deep learning structure

**Fig. 2:**
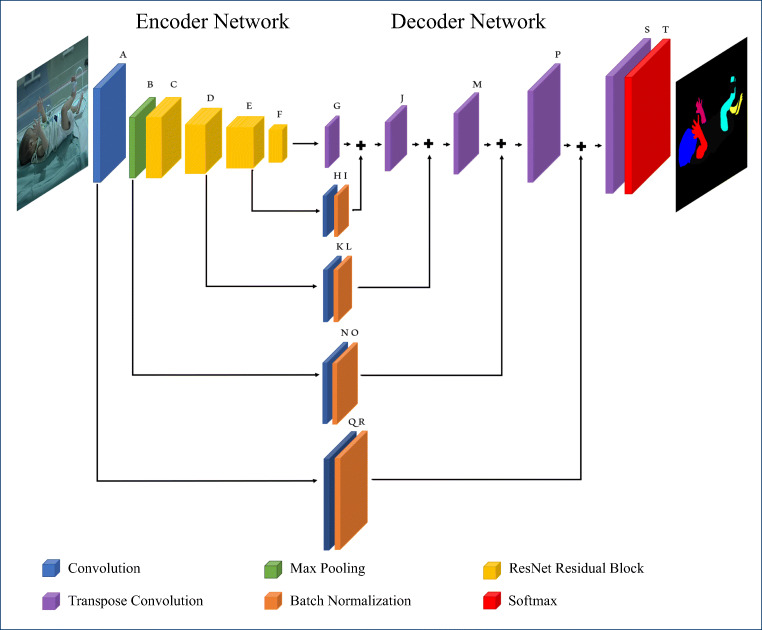
Proposed encoder-decoder structure based on the architecture proposed by Olivera et al. [21] with a modified version of the ResNet-50 [26] as encoder. Rectified linear units (ReLUs) are omitted for reasons of brevity

Figure 2 shows the proposed encoder-decoder structure. The encoder network to the left (A–F) receives an input image and outputs a rich multidimensional feature representation. On the right side, the decoder network (G–S) gradually recovers shapes and detail information from the coarse feature representation extracted from the encoder network. The output of the decoder network is a prediction mask with the same resolution as the input image. Finally, the softmax layer (T) outputs a probability map for each pixel and class. For the encoder network, a modified version of the ResNet-50 [26] pre-trained on ImageNet dataset [27] is used. For the decoder network, an architecture inspired by the one proposed by Olivera et al. [21] is used. Four variants of our network are evaluated:

- In “bilinear,” the transposed convolutional layers (Fig. 2) are replaced by a bilinear interpolation layer, i.e., no learning is involved.
- The “unconnected” variant does not include the concatenation of intermediate encoder network feature maps in the decoder network. In this decoder variant, no structural information will be harnessed from the encoder network, meaning that the upsampling process will rely exclusively on learned multi-dimensional upsampling kernels.
- In “dropout,” the batch normalization layers of the encoder networks are replaced with dropout layers. This approach is similar to the decoder network proposed by Oliveira et al. [21].
- Finally, “batchnorm” constitutes the model as depicted in Fig. 2, i.e., every convolutional layer is followed by a batch normalization layer.

### Data augmentation and transfer learning

As described above, the size of the training data used is small compared with other datasets used in deep learning scenarios. To overcome the associated challenges, data augmentation and transfer learning is used.

#### Regular augmentation

To improve the generalization of the network’s parameters, the training data was synthetically modified using standard augmentation techniques [28]. For all random operations, a uniform distribution was used:

- Scaling: since distance between camera and neonate may vary, it is important for the model to be invariant to different body scales. Thus, each training image was randomly resized by a scale factor between 0.7 and 1.4. After scaling, the images were randomly cropped/placed on a black background depending on their increase/decrease in size.
- Rotation: to increase the network’s robustness to camera rotations, each training image was randomly rotated by an angle of up to 30^∘^.
- Flipping: as the human body is symmetric, it is acceptable to randomly flip the training image horizontally.
- Color variations: to increase robustness to variations in illumination and skin color, brightness, contrast, and saturation were slightly modified within a range of 0.9 to 1.1.

In the training process on the public datasets, only scaling/cropping and rotation was used for data augmentation.

#### Pre-training/transfer learning

While the size of the annotated dataset is comparable with those found in other medical publications, it is still far smaller than those typically used in other deep learning scenarios. Since this problem often occurs in medical engineering, transfer learning might be used to overcome the challenges associated with smaller datasets. In this work, pre-training using similar datasets, namely the PASCAL human parts [29] and the Freiburg sitting dataset [21], were used. These datasets contain a great variety of body scales and poses, allowing the generation of a flexible and general initial model. Since the data is publicly available and thus poses no special demands on data security, a cloud-computing service (Google “colab” Colaboratory) was used for the pre-training. A total of 3583 images from the PASCAL dataset and 200 images from the Freiburg dataset were scaled to 320 × 320 pixels and used in the process.

#### Virtual NIR data

As argued above, imaging in the NIR domain offers less obtrusive means of illumination. However, as the images are monochromatic, no color-based skin segmentation is possible. At the same time, the available datasets used for pre-training are only available in the visual (RGB) domain. Hence, the need for annotated virtual NIR data arose. To obtain it, first, only the red channel of the RGB image was used, as it is closest to the NIR images from a spectral point of view. Next, histogram matching was performed to generate virtual NIR images that exhibit histograms more similar to real NIR data. For this, the MATLAB function “imhistmatch” is used. In short, it:

- calculates the target histogram *c*_NIR_ of the NIR data,
- calculates the actual histogram *c*_R_ of the red channel data, and
- minimizes $\left |\hat {c}_{\mathrm {R}}(T(k)) - \hat {c}_{\text {NIR}}(k)\right |$.

**Fig. 3:**
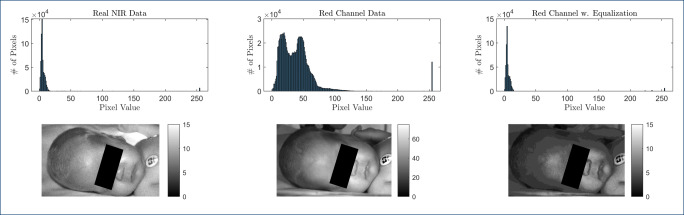
Left: Real NIR data. Center: Red channel of RGB image. Right: Virtual NIR image. The top row shows histograms; the bottom row excerpts of the corresponding images. Note the different scale of the colorbar in the center image

Here, $\hat {c}$ are the cumulative histograms at intensity *k*. *T*(*k*) is the mapping function found by the algorithm. *T* must be monotonic and $\hat {c}_{\mathrm {R}}(T(a))$ cannot overshoot $\hat {c}_{\text {NIR}}(a)$ by more than half the distance between the histogram counts at *a*. The result of the process is visualized in Fig. 3. The histogram matching was performed by matching the histogram of the red-channel images of one subject to one NIR frame of the same recoding. Thus, no information from other folds of the dataset is leaked in the process. Note that Fig. 3 also indicates underexposure of the real NIR data.

#### Implementation details

The network was implemented in Python using PyTorch. The “Adam” optimizer [30] was used for training. The initial learning rate was set to 0.0001, and the other parameters were left to their default values (β_1_ = 0.9, β_2_ = 0.999, *𝜖* = 1e − 8). In the pre-training stage, the learning rate was kept constant, while the learning rate was reduced by a factor of 0.5 every 30 epochs when training with the clinical data. The cross-entropy function was used as loss function.

## Results and discussion

In the following, the proposed approach is analyzed in terms of complexity and computational cost as well as segmentation performance.

### Complexity and computational cost

**Fig. 4:**
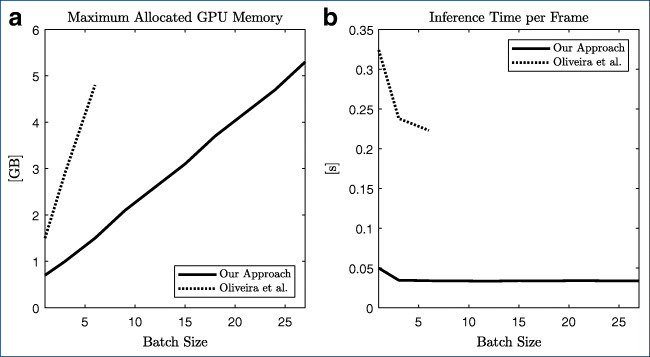
**a** Maximum allocated GPU memory **b** Inference time per image with varying batch size

As described above, our approach is based on works by Oliveira et al. However, instead of using the VGG16 network, ResNet-50 was used as an encoder. Hence, complexity and thus the number of tunable parameters as well as the number of floating-point multiply-add operations (FMAs) is reduced significantly. The differences are listed in Table 2. Additionally, Fig. 4 compares the peak memory usage during inference as well as the inference time on a NVIDIA Quadro P4000 GPU of our method to the approach proposed by Oliveira et al.

**Table 2:** Comparison of the number of tunable parameters and the number of floating-point multiply-add operations (FMAs) of the Oliveira-model and our approach

		Oliveira et al.	Our model
Number of parameters	Total	134 729 180	23 577 892
	Encoder	134 260 544	23 508 032
	Decoder	468 636	69 860
Computational complexity (GFMAs per forward pass)	Total	129.17	8.44
	Encoder	129.11	8.40
	Decoder	0.06	0.04

As one can see, the proposed modifications significantly reduce the number of free parameters as well as computational complexity. The proposed FCNN model has 18% of the learnable parameters of the encoder-decoder architecture proposed by Oliveira et al. [21]. This substantial difference in the model’s complexity mainly derives from the employed encoder network: the proposed model and its decoder variants rely on a modified ResNet-50, which is significantly smaller in the number of learnable parameters when compared with the modified VGG-16 in the Oliveira et al. architecture. The substantial increase of FMAs in the Oliveira et al. encoder-network with respect to the FMAs of the regular VGG-16 (31.51 GFMAs) derives from the padding increase (1 to 100) in the first convolutional layer of the network.

Note that inference time is also reduced in the original approach as the batch size is increased (Fig. 4b). However, due to limitations in GPU memory, 6 was the maximum batch size for inference of images with a size of 960 × 576 pixels using the Oliveira-approach (see also Figure 3a). No significant increase in inference time was observed for batches larger than 3 using our proposed approach. As expected, the allocated GPU memory increases linearly with the batch size (Fig. 4a). To conclude, the proposed method can inference 30 images per second, making it feasible for real-time segmentation applications.

### Segmentation performance

The performance was evaluated in terms of the metrics intersection over union (IoU) and accuracy (ACC) for the six segmented classes. To decide on the final implementation, the four proposed architectures were first evaluated on a combination of the PASCAL human parts dataset and Freiburg sitting images dataset. The datasets were used both unaltered (i.e., as RGB color data) and in a version converted to grayscale. After the final implementation was determined based on the external dataset, the performance of the algorithm was evaluated on the neonatal dataset. For the large external datasets, 90% of the datasets were used for training, and the remaining data was used for evaluation. As the neonatal dataset was comparatively small, 5-fold cross-validation was used to assess segmentation performance.

#### PASCAL human parts dataset and Freiburg sitting images dataset

Table 3 lists the results. The first four rows present results of different architectures on the original RGB dataset.

**Table 3:** Quantitative results on the PASCAL human parts and Freiburg sitting validation dataset

		IoU (%)						Accuracy (%)							
	Network structure variant	Head	Torso	Right arm	Left arm	Right leg	Left leg	Head	Torso	Right arm	Left arm	Right leg	Left leg	Mean IoU (%)	Mean accuracy (%)
RGB data	Bilinear	66	55	18	12	29	33	73	71	24	12	31	41	36	50
	Unconnected	47	52	10	12	30	32	51	72	10	12	30	39	31	43
	Dropout	65	53	33	32	44	43	73	67	39	40	48	46	45	63
	Batchnorm	67	56	35	36	46	45	72	69	40	43	52	52	48	66
GS data	Dropout	61	50	31	30	43	44	67	63	37	36	46	50	43	60
	Batchnorm	63	52	17	15	29	32	71	65	11	14	34	32	35	45

For each method, IoU and accuracy for each class as well as mean IoU and mean accuracy are reported

**Fig. 5:**
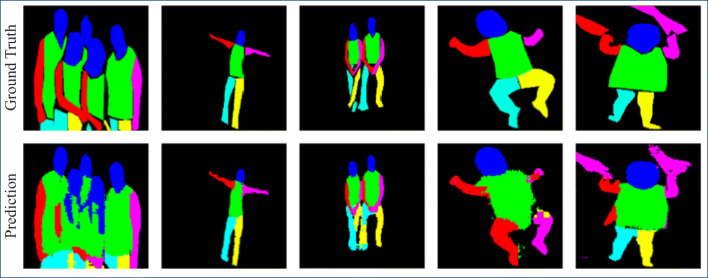
Qualitative results of the proposed “batchnorm” model on the PASCAL human parts and Freiburg sitting people dataset

The proposed encoder-decoder architecture “batchnorm” outperforms all the other methods in most of the body part classes for the RGB data. Particularly noteworthy are the significant improvements in both accuracy and IoU for thinner classes such as the right and left arm and right and left leg. Figure 5 shows qualitative results of the proposed architecture. The model shows good segmentation results on unseen testing images. However, the model has some difficulties in segmenting infants. On average, the “dropout” variant of our system resulted in the second best performance. Thus, these two architectures are selected for evaluation with grayscale data. The last two rows of Table 3 show segmentation results for the architectures when the dataset is converted to grayscale.

A marked decrease in performance could be observed for the grayscale data. This was expected as no information is available which would allow segmentation based on skin color. Interestingly, the classes “head” and “torso” are affected comparatively little. While the “dropout” network outperforms the “batchnorm” variant of the network architecture on average, it shows inferior results for the classes “head” and “torso” both in terms of IoU and ACC.

For a numeric comparison, the “batchnorm” variant was tested against the original algorithm of Oliveira et al. on the PASCAL human parts RGB dataset. Table 4 shows the condensed results. As one can see, the architecture proposed by Oliveira et al. outperforms our approach in terms of accuracy and intersection over union. While the differences are particularly noteworthy for the finer structures of arms and legs, they are less pronounced for head and torso. In the light of future real-time applications and because of the significantly reduced amount of free parameters, the “batchnorm” variant of our algorithm was decided for evaluation on the clinical dataset.

**Table 4:** Quantitative comparison of our method and the approach by Oliveira et al. on the PASCAL human parts RGB dataset

	Individual IoU (%)				Mean	
Method	Head	Torso	Arms	Legs	IoU (%)	Accuracy (%)
Batchnorm	65	55	18	36	44	53
Oliveira et al.	83	79	74	77	78	86

#### NICU data

**Fig. 6:**
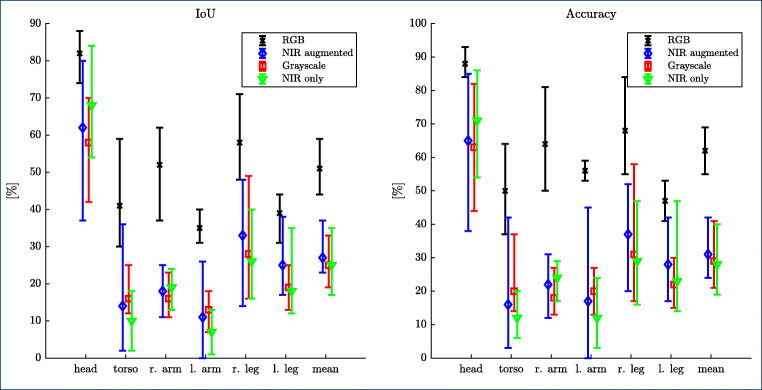
Quantitative results of the proposed CNN model “batchnorm” on the NICU dataset for the 5-folds. The training and validation images are downsampled by a factor of two in both dimensions (i.e., 960 × 576) using bilinear interpolation. The bars show minimum, mean, and maximum value

**Fig. 7:**
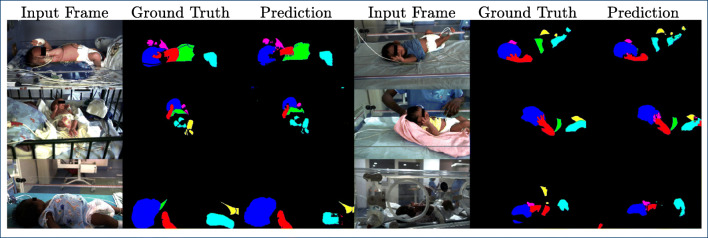
Qualitative segmentation results for the RGB data. Images were individually optimized (brightens, contrast, white balance) for visualization purposes. Note that the caregivers’ hands are ignored by the algorithm

**Fig. 8:**
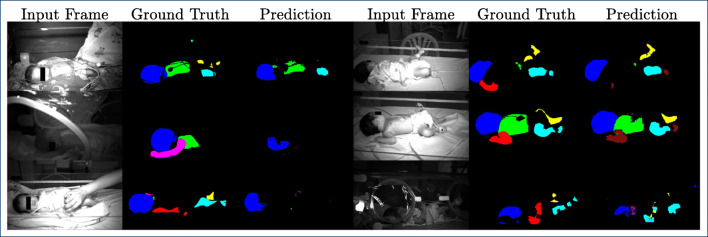
Qualitative segmentation results for the NIR data. Images were individually optimized (brightens, contrast) for visualization purposes. Note the relatively inhomogeneous illumination

In Fig. 6, quantitative results for the NICU dataset are presented for RGB, NIR, and grayscale (GS) data obtained from the histogram-matched red-channel data. Moreover, Figs. 7 and 8 give an qualitative visualization of the segmentation performance.

Several observations can be made. For the RGB data, results on the clinical data are comparable with those obtained on the PASCAL human parts dataset and Freiburg sitting images dataset. In particular, mean IoU (51%) and mean accuracy (62%) are in the range of the public dataset, 48% and 66% respectively. On average, the head is particularly well detected (IoU 82%, Acc. 88%), which is not the case for the torso (IoU 41%, Acc. 50%). One possible explanation is that in neonates, the head is relatively large and thus easier to detect, while the torso is often (partly) covered with a variety of clothing and/or cables (see also Fig. 7). In the current approach, the segmentation labels for left/right arms and legs were not flipped in the data augmentation process of flipping the input images. This may have decreased performance to some extend and needs to be analyzed in future iterations.

For the NIR data with data augmentation, results are inferior, with a mean IoU of 27% and a mean accuracy of 31%. However, the head is comparatively well detected (IoU 62%, Acc. 65%), which is again in the range of performance for the public dataset converted to grayscale. This is also obvious in the qualitative display in Fig. 8. While we are optimistic that an IoU > 50% will allow the extraction of a cardiac-related signal from the head region using NIR data, this needs to be analyzed in future work.

As the performance degraded significantly when switching from RGB to NIR, the question arises how much of this degradation can be attributed to the switching from RGB (3 channel) to grayscale (1 channel) and how much is caused by the usage of NIR data instead of RGB data. One can speculate that differences in spectral properties or a non-ideal histogram matching process could lead to this degradation. Thus, an experiment was performed where the network trained for NIR data prediction was applied to the histogram-matched red-channel data. As the network was trained with approximately 7-times as many histogram-matched frames than with real NIR frames, one would expect that the performance increases. Figure 6 shows, however, that this is not the case. In fact, performance is slightly decreased, but within the range of variation, one would expect with this relatively small dataset. Together with the observations made on the grayscale versions of the public dataset (Table 3), this indicates that the lack of color information is the major cause of performance degradation. We can further speculate that the inhomogeneous illumination as visible in Fig. 8 contributes to the greater variability in segmentation performance for some body parts such as torso and left arm. Whether or not an improved exposure will improve segmentation performance remains to be analyzed with new, gain-adjusted measurements.

**Fig. 9:**
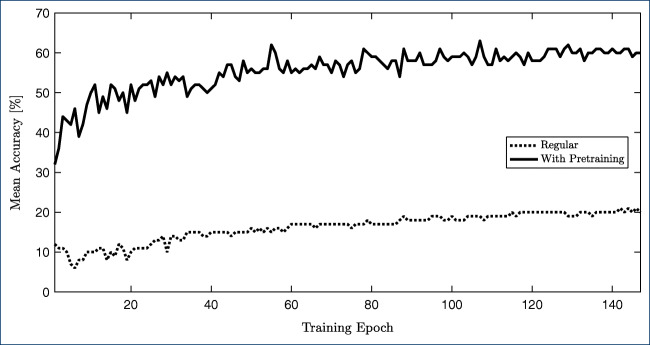
Mean accuracy over the number of epochs for the model trained with neonatal data only (regular) and when using the public dataset (with pre-training)

As described above, the public datasets were used to pre-train the model. For each fold, the pre-trained model was additionally trained with the hand-annotated data of the other folds. In Fig. 9, the effects of this transfer learning are visualized. Several beneficial effects can be observed. For one, the starting performance is higher. Next, the learning slope is higher as well; thus, convergences are achieved faster. Most strikingly, however, the final performance is significantly better: If we only used our own hand-annotated dataset, a mean accuracy of 20% is achieved, while using pre-training, this value improves to 60%. This is particularly noteworthy as the annotations on the public dataset include clothing, whereas our target annotations only included exposed skin.

If data augmentation with virtual NIR data is omitted (“NIR only” in Fig. 6), the average performance decreases. However, the decrease is relatively small, and the results for the head are actually better without the augmentation step. This further shows that our model, when pre-trained with the public dataset, only needs very few images (here only 80 NIR images in total) for finetuning.

By using the standard data augmentation described above on our dataset, no gains in performance are achieved. In fact, if we omit these steps, the performance actually increased slightly. We assume that due to our standardized setup, the data exhibits relatively little variability (in particular distance from the camera) and does not benefit from classical augmentation. Nevertheless, since it will make the model more robust for future applications, the results presented are based on training including data augmentation. To determine the limits of the proposed approach to generalize, evaluation on additional datasets with a less standardized setup will be necessary in future work.

A different observation is made in terms of the data augmentation of the NIR data via histogram equalization. Here, results improve dramatically: If we use the red channel of our data without histogram equalization, the values of IoU/accuracy drop dramatically, both for mean (17%/18%), and for the head ROI (47%/47%).

Comparing the results achieved on the public datasets with those achieved on neonatal images in terms of RGB, NIR, and grayscale data, our key learnings can be summarized as follows. For one, NIR data seems to be no less suitable than other types of monochromatic images for detecting and segmenting humans in images. Whether or not this accuracy will be sufficient, and if the differences in terms of light-tissue interaction will have an influence on the extracted cardiac signal has to be analyzed in future work. For another, the head can be segmented relatively well, even in NIR/grayscale data. We suspect that this stems from the fact that the head is relatively large in neonates, has a distinct shape, and has distinct features. This is particularly noteworthy as PPGI is extracted with great success from the head in adults.

## Conclusion and outlook

In this paper, we presented a deep learning-based method for segmentation of neonatal video data based on an architecture proposed by Olivera et al. [21] with a modified version of the ResNet-50 [26] as encoder. This reduced the computational time by a factor of 7.5, so that 30 frames per second can be processed at 960 × 576 pixels. Thus, the method is capable of performing segmentation in future real time for PPGI applications. While the presented computations were performed on expensive server-grade GPU hardware (NVIDIA Quadro P4000), we are confident that future embedded computing systems will be powerful for actual real-time implementation. Our work also continues the works of Chaichuleea et al. [9, 16], whose DL approach provided three bounding boxes at 10 frames per second.

In terms of segmentation accuracy, our approach presents promising results on the RGB data from 29 neonates recorded in two NICUs in Germany and India. In particular the head is segmented well, and performance is comparable with those achieved on large, public, non-neonatal datasets. While it took considerable effort to generate a hand-annotated dataset with 643 manually segmented frames, this amount of data seems insufficient when training the network from scratch. Only if large public datasets with similar content (segmented adults, PASCAL human parts dataset [20] and Freiburg sitting images dataset) are used for pre-training, results improve dramatically even though data (adults vs. neonates) as well as annotations (at least partial clothing vs. naked skin) were dissimilar. This is an important observation for medical DL applications in general, as the availability of data is usually limited in these settings.

While results on the RGB data were promising, performance on the NIR data was inferior. By employing data augmentation in terms of histogram equalization of the red color channel, results could be improved. Nevertheless, only the head could be segmented with satisfactory quality. In the future, experiments have to show whether or not the segmentation accuracy will be sufficient for PPGI extraction.
